# Decoding Isoprenoid Transcript–Metabolite Interactions in Carotenoid Tomato Fruit Mutants Uncovers Novel Metabolic Cross-Links

**DOI:** 10.3390/ijms27104412

**Published:** 2026-05-15

**Authors:** Sarah Frusciante, Olivia Costantina Demurtas, Giulia Falcone, Giovanni Giuliano, Gianfranco Diretto

**Affiliations:** 1 Casaccia Research Centre, Department for Sustainability, Italian National Agency for New Technologies, Energy and Sustainable Development (ENEA), Via Anguillarese 301, 00123 Rome, Italy; olivia.demurtas@enea.it (O.C.D.); giovannigiuliano1@gmail.com (G.G.); 2 College of Horticulture, Northwest A&F University, Yangling 712100, China

**Keywords:** tomato, carotenoid biosynthesis, fruit ripening, isoprenoids, metabolomics, transcriptomics, correlation analysis

## Abstract

Carotenoids are an important class of natural compounds, essential for human nutrition, acting in plants as pigments and apocarotenoid precursors. Tomato is a key model for carotenoid metabolism, as genetic variation strongly affects carotenoid composition during fruit ripening. To date, most of the enzymes involved in the carotenoid pathway were mainly characterized by linking gain- or loss-of-function phenotypes to their genetic basis (e.g., mutation in a single gene), with limited integration into pathway-wide analyses. Here we report an extensive biochemical and molecular characterization of a collection of tomato carotenoid mutants—*apricot* (*at*), *yellow flesh* (*r*), *tangerine* (*t*), *Delta* (*Del*) and *Beta* (*B*)—throughout three different stages of fruit ripening (mature green, breaker, red ripe). Using correlation-based integrative analyses, we integrated targeted isoprenoid metabolomics (carotenoids, chlorophylls, tocochromanols, quinones, abscisic acid) with gene expression profiling and correlation-based analyses. The pronounced, stage-dependent remodeling of the isoprenoid profiles exceeded the expected changes in substrates/products and was accompanied by significant transcriptional changes, largely independent of the position of the mutated step in the pathway. This integration highlighted metabolite/transcript regulatory links and the central role of lycopene cyclization in isoprenoid metabolism rewiring, thus improving our understanding of mechanisms controlling their accumulation during tomato fruit ripening.

## 1. Introduction

Isoprenoids represent one of the most diversified classes of plant specialized metabolites, and comprise pigments like carotenoids, electron carriers like quinones and hormones like ABA that shape development and stress responses [1]. Additional plastid-localized metabolites, like tocochromanols and chlorophylls, contain an isoprenoid moiety [2,3]. Carotenoids and their cleavage products, apocarotenoids, play fundamental ecological, physiological and nutritional roles in plants and animals, including signaling mediated by apocarotenoids such as abscisic acid (ABA) or strigolactones (SLs) [4,5].

In higher plants, isoprenoids derive from two compartmentalized pathways: the cytosolic mevalonate (MVA) pathway, producing IPP from acetyl-CoA and supplying phytosterols, quinones and sesquiterpenoids; and the plastidial 2-C-methyl-D-erythritol 4-phosphate (MEP) pathway, initiated from pyruvate and glyceraldehyde-3-phosphate, which provides precursors for carotenoids, chlorophylls, tocochromanols and diterpenoids [6].

**Figure 1:**
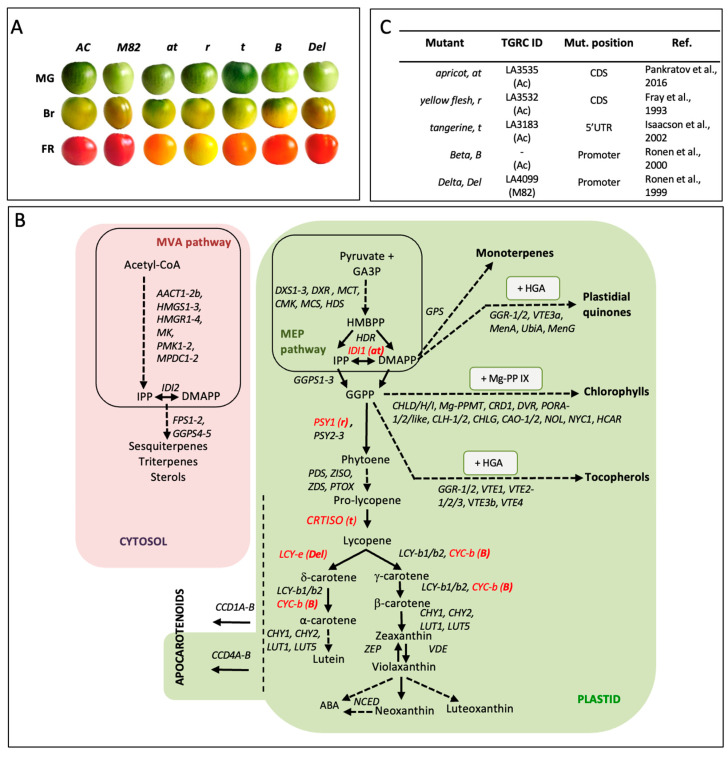
Tomato carotenoid mutants analysed in this study and their position within isoprenoid metabolism. (**A**) Fruit phenotype of wild type (AC, M82) and mutants (*at*, *r*, *t*, *B*, *Del*) across three ripening stages (MG, Br, FR). (**B**) Overview of isoprenoid biosynthesis, highlighting the carotenoid pathway within the MVA/MEP network; mutated genes are shown in red, dashed arrows indicate multistep reactions or apocarotenoid branches. (**C**) Summary of mutants, including TGRC accessions, mutation sites, genetic background, and references [11,12,13,14,15].

Tomato ripe fruits owe their red color to the massive accumulation of the unusual linear carotene lycopene, due to the upregulation and downregulation during ripening of genes for lycopene biosynthesis and utilization, respectively [7,8]. Unlike in vegetative tissues, where carotenoids are essential for oxygenic photosynthesis and thus carotenoid mutants are lethal, mutants affecting carotenoid biosynthesis in the tomato fruit are viable. Fruit-specific mutations are at least partially due to the extensive gene duplication in the carotenoid pathway, following a whole genome triplication that occurred approximately 60 million years ago, that generated fruit-specific gene paralogs that control carotenoid biosynthesis [9,10] (Figure 1). Examples of fruit-specific carotenoid mutants include *apricot* (*at*) and *yellow flesh* (*r*), carrying loss-of-function mutations in the fruit-specific isopentenyl diphosphate isomerase and phytoene synthase paralogs, *IDI1* and *PSY1*, respectively [11,12], as well as *Beta* (*B*), which carries a gain-of-function mutation in the fruit-specific lycopene β-cyclase paralog *CYC-b* [13]. In contrast, the loss-of-function *tangerine* (*t*) and gain-of-function *Delta* (*Del*) mutations alter fruit carotenoid composition but do not cause major alterations in vegetative growth, despite affecting the single-copy carotenoid isomerase (*CrtISO*) and lycopene ε-cyclase (*LCY-e*) genes [14,15]. Tomato fruit carotenoids have been extensively engineered through transgenic approaches [16,17,18]. These engineering efforts have sometimes resulted in pleiotropic effects, both at the level of accumulation of related isoprenoids like ABA [19] and at the level of gene expression in the carotenoid/isoprenoid pathways [20,21,22,23], suggesting extensive regulatory cross-talk occurs in the fruit isoprenoid pathway.

To better understand this regulatory cross-talk, we decided to study the *at*, *r*, *t*, *B*, *Del* mutants during tomato fruit ripening (Figure 1A), integrating targeted metabolomics and transcriptional profiling of carotenoids and related isoprenoids (Figure 1B) with correlation-based network analyses. This approach enabled the identification of mutation-specific regulatory patterns, metabolic modules, and previously unrecognized cross-talks between isoprenoid classes, highlighting regulatory nodes of interest for future breeding/metabolic engineering programs.

## 2. Results

### 2.1. Confirmation of the Genetic Lesions of the at, r, t, B, Del Tomato Mutants

The loss-of-function mutations in *IDI1* (*apricot*, *at*), *PSY1* (*yellow flesh*, *r*), and *CrtISO1* (*tangerine*, *t*), and gain-of-function mutation in the chromoplast-specific lycopene β-cyclase *CYC-b* (*Beta*, *B*) were in the Ailsa Craig genetic background, while the gain-of-function mutation in the lycopene δ-cyclase *LCY-e* (*Delta*, *Del*) (Figure 1B), was in the M82. For the 5 mutants preliminary genetic information were available (Figure 1C) and all the alleles were re-sequenced and confirmed to carry the previously described lesions (Figures S1–S3; Table S2), including a ∼3 kb insertion in *PSY1* (*r*), a frameshift mutation in *IDI1* (*at*), a 5′UTR deletion in *CrtISO1* (*t*), as well as extensive promoter polymorphisms in the *CYC-b* (*B*) and *LCY-e* (*Del*) alleles.

### 2.2. Transcript–Metabolite Cross-Talk in the Carotenoid Pathway

**Figure 2:**
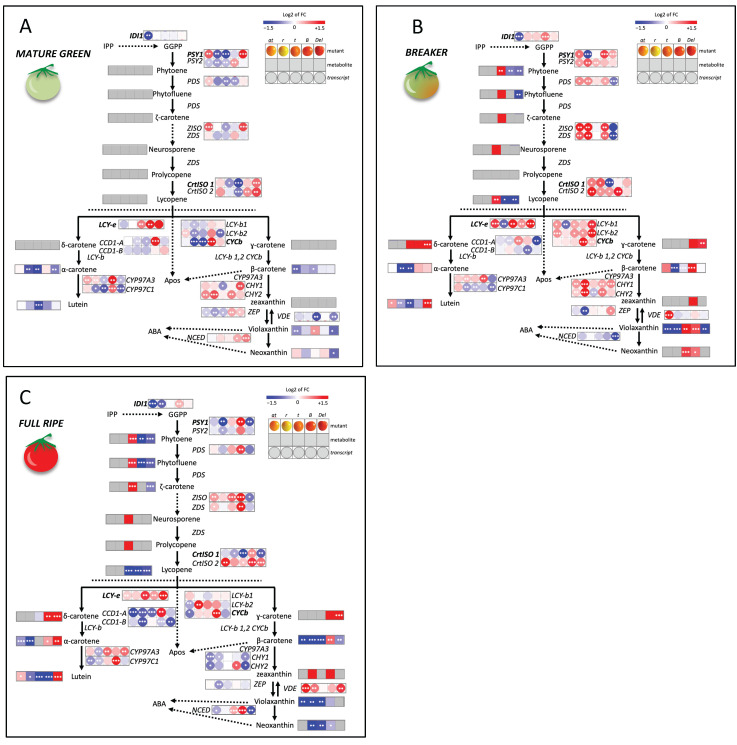
Biochemical and molecular changes, depicted using heatmap visualization, in carotenoid biosynthesis during ripening in tomato mutants at MG (**A**), Br (**B**), and FR (**C**) stages. Transcript (qRT–PCR) and metabolite (HPLC-DAD-HRMS) data are shown as log_2_ fold change relative to the corresponding wild-type (AC for *at*, *r*, *t* and *B*; M82 for *Del*). Mutants are displayed in the heatmaps according the following order: *at*, *r*, *t*, *B*, *Del*. Metabolites are represented by squares, and transcripts by circles. Red and blue indicate higher and lower relative levels vs. wild-type, respectively; white indicates no change; gray indicates not detected. Student’s *t*-test: * *p* ≤ 0.05; ** *p* ≤ 0.01; *** *p* ≤ 0.001.

Fruits at the Mature Green (MG), Breaker (Br) and Fully Ripe (FR) were subjected to targeted metabolite and transcript analyses. Across all genotypes and stages, we identified and quantified 44 carotenoids by liquid chromatography with photodiode array detector coupled to high resolution mass spectrometry (LC-DAD-HRMS) (Tables S3 and S4) and measured the abundance of 22 endogenous transcripts by Real Time-PCR (Table S5). These datasets were integrated into a custom metabolic map including all quantified metabolites and genes, plotted as wild-type-normalized values for each mutant and stage (Figure 2A–C). In broad terms, MG fruits of all mutants showed only modest changes in carotenoid profiles but already exhibited clear transcriptional reprogramming, whereas Br and especially FR stages displayed stronger and genotype-specific perturbations in both metabolites and transcripts, affecting the entire pathway. The main biochemical and molecular features of these responses are summarized below for each mutant.

At the mature green stage, minor reductions in α- and β-carotene were observed in loss-of-function lines (*at*, *r*, *t*), while the xanthophyll branch was altered in *t* and *Del*, with violaxanthin showing opposite trends between the two genotypes (Figure 2A and Table S4A). Carotenoid transcripts were more strongly perturbed: in *at*, most early pathway genes were upregulated, except for the mutated *IDI1* one. In contrast, *r* and *t* showed widespread down-regulation along the pathway, with few exceptions (*CHY2* and *LUT5* in *r*; *LCY-e* in *t*) (Figure 2A and Table S5A). Notably, the gain-of-function *B* mutants exhibited an extensive transcriptional activation of most pathway genes. This was associated with depletion of early intermediates and accumulation of downstream carotenoids, together with increased expression of apocarotenoid-related genes (*CCD1a*, *NCED*) (Figure 2A and Table S5A). Finally, *Del* displayed a distinct transcriptional profile: early genes were induced, but late genes showed mixed responses across both the ε–β– and β–β– branches (Figure 2A and Table S5A).

At the breaker stage, carotenoid profiles diverged markedly between mutants compared to MG. *Cis*- and *all-trans* lycopene were absent in *at* and *r*, and strongly reduced in *B* and *Del*, whereas *t* showed accumulation of *cis*-lycopene and early carotenoids, consistent with previous observations [14] (Figure 2B and Table S4B). β–β– xanthophylls (violaxanthin, neoxanthin) were decreased in early *r* and *at*, with a compensatory increase of lutein in the latter mutant. δ-carotene accumulated in *Del*, but also, unexpectedly, in *B* and *t*, a previously unreported feature [13,14]. *Del* accumulated lutein but unexpectedly, also γ-carotene. Similarly, *B* accumulated viola- and neoxanthin (Figure 2B and Table S4B).

Transcriptional perturbation at Br exceeded that observed at MG (Figure 2B and Table S5B). In *at*, a generalized pathway activation involved both early and late genes, including strong induction of desaturation/isomerization steps and lycopene cyclases, suggesting compensation for the upstream bottleneck. In *r*, early genes were strongly induced whereas several downstream genes remained repressed, amplifying the MG pattern; carotene hydroxylases were induced, consistent with enhanced lutein accumulation. In contrast, *t* showed partial normalization of gene expression, with most MG differences attenuated, indicating that its Br phenotype is primarily metabolic rather than transcriptionally driven.

The *B* mutant maintained strong pathway-wide activation, with *CYC-b* reaching very high expression levels, supporting a positive feedback mechanism reinforcing β–β– flux. In *Del*, transcriptional changes were moderate and selective: early genes were slightly induced, several β-branch transcripts repressed, and *NCED* reduced, consistent with metabolic rerouting toward the ε–β–branch (Figure 2B and Table S5B).

As expected, fully ripe fruits showed the strongest divergence from wild-type, reinforcing Br trends while revealing late-stage shifts in branch usage (Figure 2C and Table S4C).

In *at*, early intermediates (from phytoene to lycopene) remained absent, α- and β-carotene were reduced, and lutein stayed elevated, indicating persistent rerouting toward the ε-branch despite the upstream bottleneck. In *r*, early intermediates were still undetectable, and most carotenoids were strongly depleted; notably, zeaxanthin appeared de novo at FR. In *t*, early carotenoids accumulated strongly, with prolycopene as the hallmark of impaired *CrtISO1* activity. Upstream intermediates such as neurosporene and ζ-carotene increased, and both *cis* and *all-trans* lycopene remained detectable. Downstream carotenoids, including xanthophylls, were broadly reduced, although total carotenoid content was not markedly decreased. In *B*, phytoene, phytofluene and lycopene were strongly reduced and total carotenoids declined to ~50% of wild-type, indicating upstream precursor limitation. Nevertheless, the β–β– branch remained highly active (high β-carotene and detectable zeaxanthin), while the β–ε– branch also increased (δ-carotene and α-carotene). Furthermore, γ-carotene and δ-carotene persisted, confirming stable accumulation of unusual intermediates (Figure 2C and Table S4C). In *Del*, early intermediates decreased but total carotenoids declined only modestly. Lycopene and β-carotene were sharply reduced, whereas δ-carotene, γ-carotene and α-carotene increased and lutein remained strongly elevated, consistent with sustained ε–β– activation and restricted β–β– flux (Figure 2C and Table S4C).

Gene-expression profiles paralleled metabolic outcomes (Figure 2C and Table S5C). In *at*, several early genes (*Z-ISO*, *ZDS*, *CrtISO2*) remained upregulated, while mutated *IDI1* stayed reduced. *LCY-e* increased, whereas β-branch transcripts (*LCY-b2*, *CHY1*/*2*, *LUT5*, *NCED*) were consistently downregulated. In *r*, *PSY1*/*PSY2* (and *IDI1*) were reduced together with most late genes, except for *LCY-b2* and *VDE* which were induced. In *t*, the mutated *CrtISO1* was further reduced (together with *CCD1a*), most early genes remained close to wild-type levels, and only a limited subset (*Z-ISO*, *CrtISO2*, *LCY-e*, *LUT1*) was induced; conversely, *NCED* increased. In *B*, *CYC-b* reached extremely high levels and was accompanied by induction of early desaturation/isomerization genes (*PSY1*/*2*, *PDS*, *ZISO*, *ZDS*, *CrtISO1*/*2*) and β-ring hydroxylases (*CHY2*, *LUT5*); *CCD1a* and *NCED* increased, whereas *CCD1b* decreased. Finally, in *Del* the early genes *PSY1*, *Z-ISO*, and *CrtISO1* were reduced, while *LCY-e* and *LUT1* remained upregulated, whereas β-branch genes (*LCY-b* isoforms, *CHY1*, *CCD1b*) and *NCED* were downregulated (Figure 2C and Table S5C).

### 2.3. Transcriptional–Metabolic Remodeling of ABA Pathways

**Figure 3:**
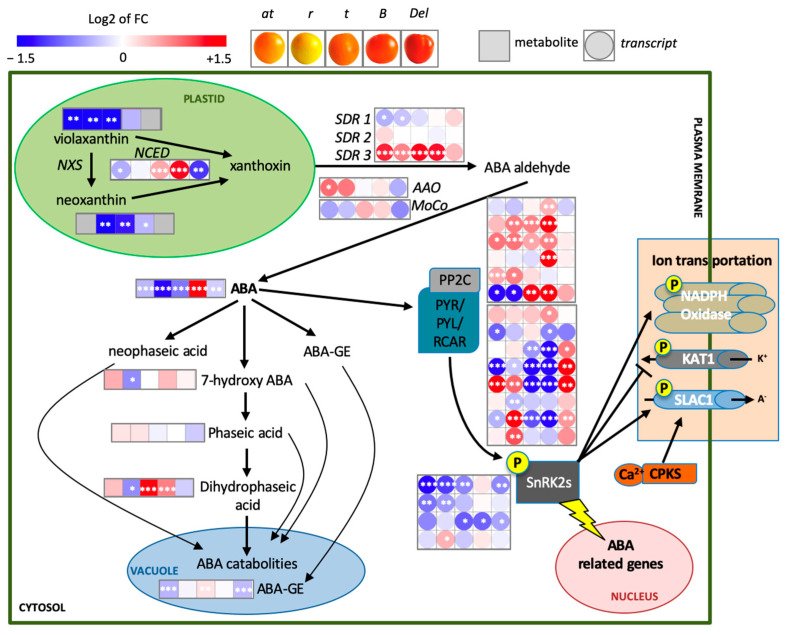
ABA biosynthesis and related changes in tomato mutants at the full ripe (FR) stage. Transcripts and metabolites data are shown as log2 fold change relative to the appropriate wild-type (AC for *at*, *r*, *t* and *B*; M82 for *Del*). Red and blue indicate higher and lower relative levels vs. wild-type, respectively; white indicates no change; gray indicates not detected. Student’s *t*-test: * *p* ≤ 0.05; ** *p* ≤ 0.01; *** *p* ≤ 0.001. See Section 4 and Tables S6 and S7 for details.

ABA is a direct downstream product of carotenoid metabolism and plays a central role in fruit physiology [5]. We profiled ABA-related metabolites by LC-ESI-HRMS and integrated these data with EU-TOM3 [19] transcript profiles into a custom pathway map (Figure 3 and Figure S4; Tables S6 and S7). At MG and Br, ABA changes were modest and genotype-specific, with minor and inconsistent transcriptional variation (Figure S4 and Tables S6 and S7). In contrast, FR fruits displayed a clear divergence: ABA decreased in *at*, *r*, *t*, and *Del*, but strongly increased in *B*, consistent with enhanced β-branch flux. Transcriptional responses mirrored these trends, with *at* and *t* showing coordinated down-regulation of ABA-related genes, *r* and *Del* activating PYL- and WRKY-mediated responses, and *B* inducing *PP2C* genes, indicative of reinforced ABA signaling (Figure 3 and Tables S6 and S7).

### 2.4. Remodeling of Other Isoprenoid Pathways

**Figure 4:**
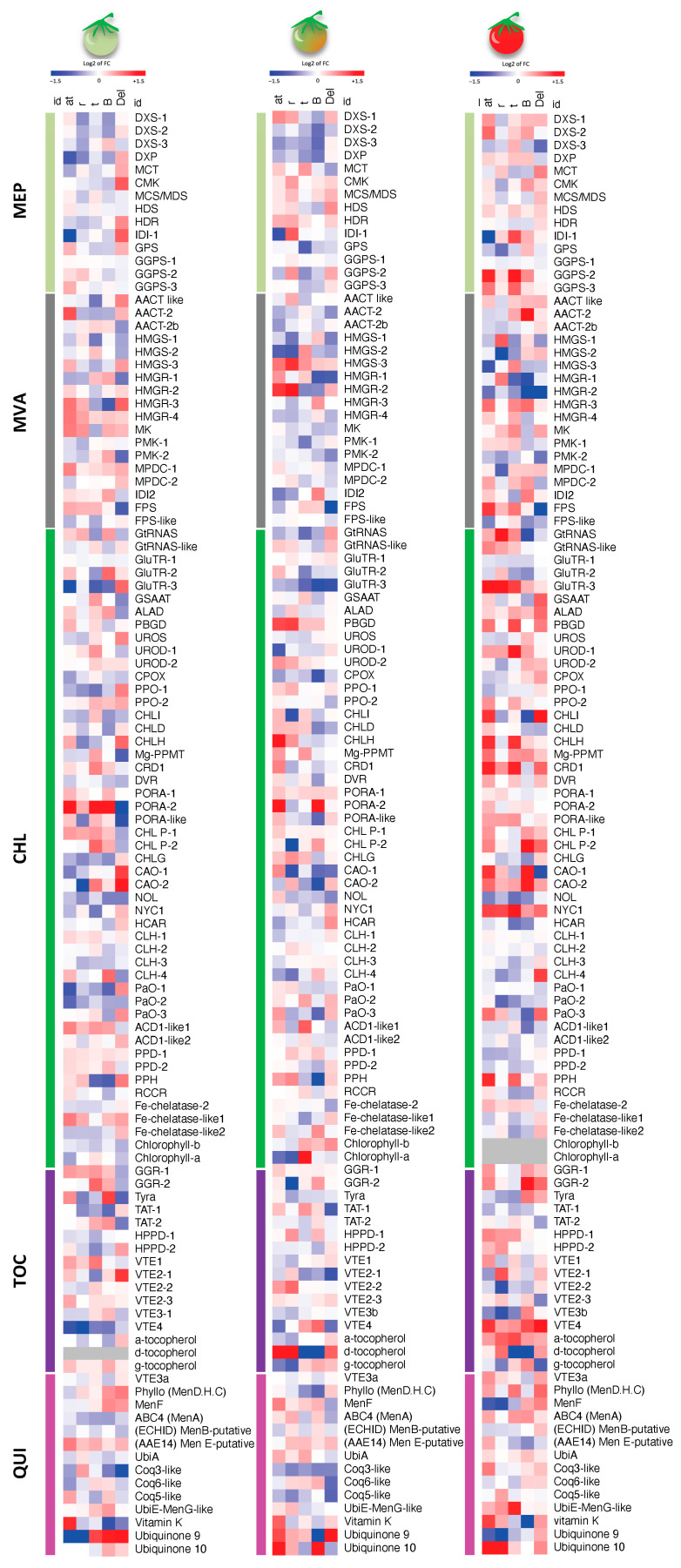
Heatmaps of isoprenoid-related transcripts and metabolites in tomato mutants during ripening. Transcript and metabolite data from carotenoid-associated pathways (MVA, mevalonate; MEP, methylerythritol 4-phosphate; CHL, chlorophylls; TOC, tocochromanols; QUI, quinones) are shown for MG, Br, and FR as log_2_ fold change relative to the corresponding wild-type (AC for *at*, *r*, *t*, *B*; M82 for *Del*). Color scale: red, higher; blue, lower; white, unchanged; gray, not detected. See Section 4 and Tables S8 and S9 for details.

To assess the broader impact of carotenoid perturbations on isoprenoid metabolism, we analyzed transcript profiles of the plastidial MEP and cytosolic MVA pathways, along with chlorophyll, tocochromanol, and quinone pathways. Representative metabolites (chlorophylls a/b, tocopherols, phylloquinone, and ubiquinones-9/10) were quantified and integrated with transcript data as wild-type normalized heatmaps across MG, Br, and FR stages (Figure 4 and Tables S8 and S9).

#### 2.4.1. MEP/MVA Pathways

Transcriptional changes in the MEP and MVA precursor pathways were generally modest at MG and Br but became more evident and genotype-specific in FR fruits (Figure 4 and Table S9). Overall, the response was not coordinated at the whole-pathway level, while involved selective changes in specific nodes of the two pathways. In *at*, FR fruits showed induction of *DXS-1*, *DXS-2* and *GGPS-2*/*3*, whereas *IDI1* was strongly reduced. In *r*, changes were comparatively moderate and scattered across both pathways, without clear coordinated repression. In *t*, the most evident responses were the induction of *IDI1* and *GGPS-2*, while most other MEP/MVA transcripts showed limited or mixed variation. In *B*, transcript changes were also heterogeneous, with induction of selected genes such as *AACT-2* but reduction of several MVA transcripts, including *HMGR* isoforms. Likewise, *Del* displayed a mixed pattern, with repression of some MVA genes, particularly *HMGR* isoforms and *FPS*, alongside limited or variable changes in other MEP/MVA transcripts (Figure 4 and Table S9).

#### 2.4.2. Chlorophyll Biosynthesis

Chlorophyll metabolism was only weakly affected (Figure 4 and Tables S8 and S9). As expected, chlorophylls declined during ripening and were undetectable at FR in all genotypes. At earlier stages, only at (reduced chlorophyll a at MG/Br) and t (increased chlorophyll a/b at Br) showed consistent metabolic differences. Transcriptomic changes were generally modest and isoform-specific, with no coherent genotype-driven pattern. Although differential expression increased at FR, these changes did not translate into measurable pigment differences, suggesting limited biological impact relative to carotenoid remodeling.

#### 2.4.3. Tocochromanol/Quinone Pathways

Tocochromanol and quinone pathways were moderately affected by carotenoid perturbations, with most changes being genotype- and stage-dependent rather than coordinated at the pathway level (Figure 4 and Tables S8 and S9). At MG and Br, transcript changes were generally limited and often isoform-specific, whereas metabolite profiles already showed some mutant-dependent variation. In FR fruits, the clearest effects were observed at the metabolite level. α-Tocopherol increased in all mutants, while γ-tocopherol showed a more restricted response and accumulated mainly in *B.* In contrast, δ-tocopherol displayed opposite behaviours among genotypes, increasing in *at* and *r* but decreasing in *t* and *B* (Figure 4 and Tables S8 and S9). Quinone metabolites were likewise variably affected. Phylloquinone/vitamin K showed clear genotype-dependent changes, increasing in *at* and *Del* but decreasing in *B* at FR. Ubiquinones-9 and -10 also displayed marked stage- and genotype-specific variation, without a consistent trend across mutants. At the transcriptional level, FR fruits showed broader but still heterogeneous remodelling. Several tocochromanol-related genes, including *VTE4*, were induced in multiple mutant backgrounds, whereas quinone-related transcripts remained variably affected and did not reveal coordinated pathway-wide regulation (Figure 4 and Tables S8 and S9). Overall, transcriptional shifts only partially matched metabolite outputs, suggesting substantial buffering at the metabolic level.

### 2.5. Integration of Metabolite–Transcript Data

To explore relationships across isoprenoid pathways and assess how carotenoid mutations reshape the metabolic–transcriptional landscape, we integrated metabolite and transcript datasets using stage-specific hierarchical clustering (HCL) and Pearson correlation analyses across ripening. We also constructed hub-centered correlation networks, focusing on the strongest associations around each mutated gene to evaluate how individual lesions rewire interaction patterns during ripening. These correlation-based networks capture coordinated variation between transcripts and metabolites, rather than direct regulatory interactions.

#### 2.5.1. Hierarchical Clustering (HCL) Across Ripening Stages

HCL integrating all quantified metabolites and transcripts at MG, Br and FR (Figure S5) resolved genotype similarities within a unified isoprenoid space. At MG, mutants grouped into two main pairs (*at* with *r*, *t* with *B*), while *Del* formed an independent branch, distinguishing it from the other genotypes, although this separation may partially reflect its different genetic background effects (M82). A similar topology was retained at Br, suggesting stable genotype relationships during the onset of ripening.

At FR, clustering was partially reorganized: *at* and *r* remained associated, *B* and *Del* converged into the same cluster, suggesting partial similarity at FR, although background-specific effects cannot be excluded, and *t* segregated as a distinct branch, defining a unique terminal transcriptional–metabolic state (Figure S5).

#### 2.5.2. Correlation Matrix of Carotenoid Pathway

**Figure 5:**
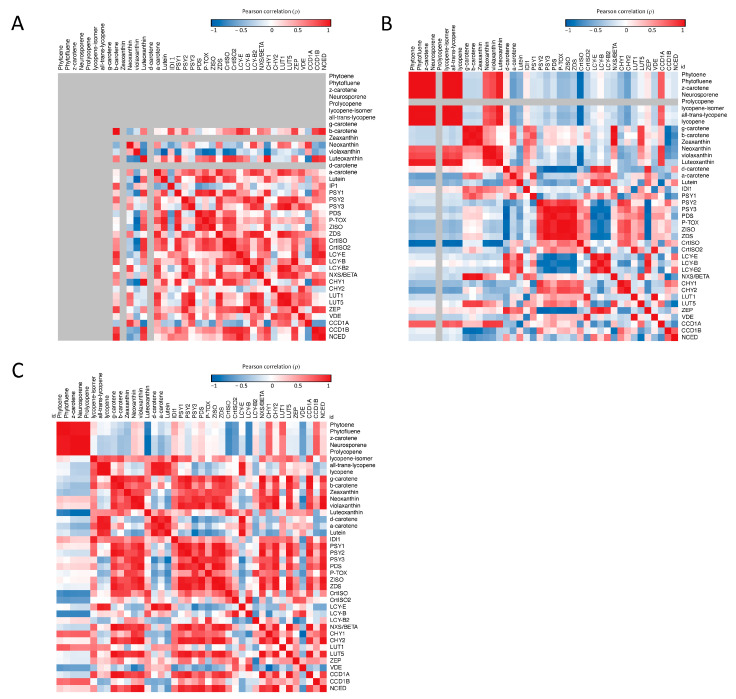
Correlation heatmaps between carotenoid-related metabolites and transcripts in tomato mutant fruits at the MG (**A**), Br (**B**), and FR (**C**) ripening stages. Each cell represents the Pearson correlation coefficient (r) between the variable in the corresponding row and column, shown as a false color scale. Correlations were computed in R and visualized with Morpheus web tool (Broad Institute, https://software.broadinstitute.org/morpheus (accessed on 11 May 2026)). See Section 4 and Tables S10–12 for details.

Pearson correlation matrices including all carotenoid metabolites and biosynthetic genes at MG, Br and FR (Figure 5 and Tables S10–12) revealed progressive restructuring of transcription–metabolite relationships during ripening. At MG (Figure 5A and Table S10), correlations reflected the profile of unripe fruit, dominated by xanthophylls and α-/β-carotene, as early intermediates were largely absent. Luteoxanthin and neoxanthin formed an inverse-correlation block relative to other carotenoids, whereas lutein, α-carotene, and β-carotene clustered together. Transcriptionally, desaturation/isomerization genes (*PDS*, *PTOX*, *Z-ISO*, *ZDS*, *CrtISO1*, *CrtISO2*) formed a compact module, with coupling between *CrtISO1* and lycopene cyclases (*LCY-b1*, *LCY-b2*, *CYC-b*), indicating coordinated early pathway activity. At Br (Figure 5B and Table S11), the matrix became more structured. A strong positive metabolite block emerged among phytoene, phytofluene, lycopene isomers, γ-carotene, β-carotene, and β–β–xanthophylls, reflecting coordinated pigment accumulation. Two opposing transcriptional modules were evident: desaturation/isomerization genes clustered together and were negatively correlated with lycopene cyclases (*LCY-b1*, *LCY-b2*, *LCY-e*). *CrtISO1* showed the strongest negative correlations with early linear intermediates, highlighting separation between precursor accumulation and downstream cyclization. At FR (Figure 5C and Table S12), the matrix became highly polarized. Metabolites segregated into two positively correlated groups: early/central intermediates and late carotenoids/xanthophylls. The ε–β– branch (δ-/α-carotene and lutein) correlated with lycopene and its isomers, indicating tight linkage to the central pool. Transcriptionally, an early-pathway module (*IDI*, *PSY1*/*2*/*3*, *PDS*, *ZISO*, *ZDS*, *CrtISO1*/*CrtISO2*) reflected coordinated regulation of upstream steps, while a second module centered on *CYC-b* (with *CHY1*/*CHY2* and *LUT5*) aligned with β-branch metabolites. *CCD1a* and *NCED* were associated with this block, linking cyclization, hydroxylation, and cleavage processes. *CrtISO1* maintained selective anticorrelation with early intermediates.

#### 2.5.3. Hub-Centered Correlation Networks Across Ripening Stages

**Figure 6:**
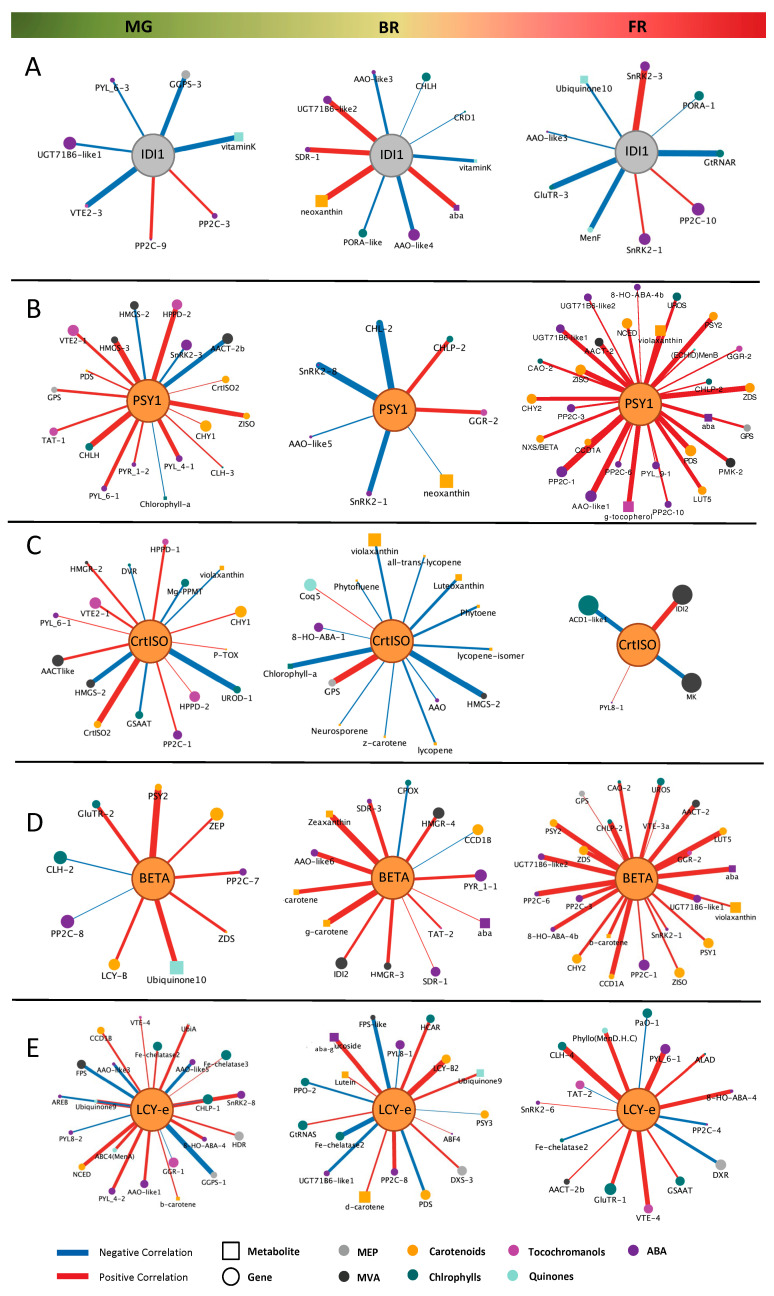
Hub-centered correlation networks of mutated carotenoid genes across ripening (MG, Br, FR). For each mutant, the causative gene (orange) is connected to features strongly correlated with its expression (Pearson |r| ≥ 0.88). Rows (**A**–**E**): *IDI1*/*at*, *PSY1*/*r*, *CrtISO1*/*t*, *CYC-B*/*B*, *LCY-e*/*Del*; columns: MG, Br, FR. Edge color indicates correlation sign (red, positive; blue, negative), edge width scales with |r|. Node size reflects node strength (ns). See Section 4 and Tables S13 and S14 for details.

To determine how carotenoid mutations reshape local metabolic organization, we constructed hub-centered correlation networks using strong Pearson correlations (|ρ| ≥ 0.88), with each mutated gene as the central node (Figure 6 and Tables S13 and S14). This approach allowed us to track mutation-specific rewiring of local interaction landscapes across ripening. Overall, network architecture was highly dynamic and genotype-dependent across ripening stages. Rather than following a common trend, individual hubs displayed distinct trajectories, ranging from expansion and densification to progressive contraction or selective rewiring.

The *IDI1*-centered network remained relatively small across stages and was predominantly composed of negative correlations (Figure 6A). At Br, it transiently connected to ABA-related components, while at FR connectivity decreased further, with only few positive associations retained, indicating a weak and unstable interaction landscape.

*PSY1* displayed strong stage-dependent rewiring (Figure 6B). At MG, it formed a highly connected hub dominated by positive associations with carotenoid and isoprenoid-related components. At Br, the network contracted and shifted toward negative correlations. In contrast, at FR, *PSY1* emerged as one of the most highly connected hubs, with extensive positive associations spanning carotenoid metabolism, ABA-related components, and upstream isoprenoid pathways, indicating broad network integration at late ripening.

The *CrtISO1*-centered network showed a progressive reduction in connectivity across ripening (Figure 6C). At MG, it displayed a mixed pattern of positive and negative correlations, including associations with genes from both plastidial and cytosolic isoprenoid precursor pathways (e.g., MEP- and MVA-related components), as well as tetrapyrrole-related transcripts. Br was characterized by strong negative associations with carotenoid intermediates. At FR, the network collapsed into a small set of correlations, indicating a marked contraction of its interaction landscape.

*CYC-b* exhibited a progressively expanding and predominantly positive network (Figure 6D). From a compact hub at MG, connectivity increased at Br and reached its maximum at FR, where *CYC-b* formed one of the most densely connected networks. Associations spanned carotenoid biosynthesis, ABA metabolism, and precursor pathways, highlighting extensive cross-talk across isoprenoid branches.

The *LCY*-e-centered network displayed a heterogeneous and stage-dependent pattern, without a clear trend of expansion or contraction across ripening (Figure 6E). At MG and Br, the network displayed a heterogeneous pattern with both positive and negative associations involving carotenoid, quinone, and tetrapyrrole-related components. At FR, connectivity decreased, resulting in a smaller but more defined set of correlations, indicating selective reorganization of its interaction network. Collectively, these results highlight genotype-specific differences in network connectivity and correlation patterns across ripening stages.

## 3. Discussion

Fruit color is the most immediate phenotypic outcome of ripening-associated carotenoid remodeling in tomato, integrating both increased carotenoid abundance and shifts in branch partitioning. Although the biochemical steps and developmental expression patterns of carotenoid enzymes are well characterized, the regulatory framework linking transcript dynamics to metabolite trajectories during ripening remains not fully resolved. This gap reflects the multilayered control of carotenoid output, extending beyond core pathway reactions. In particular, MEP precursor supply can act as a limiting node: lesions in *SlIDI1*, in fact, are sufficient to reduce carotenoid levels and alter their composition in ripe fruit [24] underscoring how precursor availability intersects with pathway-intrinsic regulation and plastid context [12].

Only a limited number of studies have directly addressed transcript–metabolite regulation within the carotenoid pathway. In green tissues, carotenoid and chlorophyll biosynthesis is tightly co-regulated, with regulation exerted at the level of carotenoid gene promoters [25]. Epistatic interactions between *CrtISO1* and *PSY1* [26], feedback effects revealed by altered desaturation capacity [27], and plastid-dependent compensation mechanisms involving *PSY2* in *r* [28], all point to non-linear and context-dependent regulation.

More recently, gene–metabolite profiling in contrasting fruit color backgrounds (red and orange) highlighted coordinated changes in *SlPSY2*, *SlPDS*, *SlZDS* and *SlCrtISO2* [29].

Together, these observations indicate that, despite extensive biochemical knowledge, the systems-level mechanisms synchronizing transcript modules with metabolite dynamics—and their reshaping by distinct genetic lesions—remain only partially understood.

Here, we combined transcriptomic and metabolomic analyses in five carotenoid mutants (*at*, *r*, *t*, *B*, and *Del*) affecting distinct steps of the pathway (Figure 1), extending the analysis to additional isoprenoid branches. Prior to integration, promoter analysis revealed additional cis-regulatory elements in *B* and *Del*, including motifs associated with light/plastid and hormone-responsive regulation (Figures S1–S3 and Table S2), consistent with altered transcriptional control of these loci [30,31,32,33,34,35].

### 3.1. Transcript–Metabolite Rewiring of Carotenoid Metabolism in Ripening Fruit

Across genotypes, integration of metabolite and transcript data across MG, Br and FR revealed frequent uncoupling between transcriptional shifts and metabolite outputs. While FR phenotypes matched expectations—severe carotenoid depletion in *at* and *r* [11,12], prolycopene accumulation in *t* [14], and β- and ε-branch enrichment in *B* and *Del* [13,15]—our stage-resolved analysis showed that pathway output reflects both transcriptional regulation and constraints imposed by precursor supply and flux redistribution.

*Apricot* (*at*)—*precursor-limited flux collapse*. In *at*, early carotenoids were essentially absent throughout ripening, indicating strong upstream limitation. Late products remained partially buffered at MG but progressively declined toward FR, consistent with the drastic carotenoid depletion reported in ripe fruits [12]. Transcriptional changes only partially paralleled metabolite loss. Together, these patterns support a model in which the phenotype primarily reflects constrained precursor input rather than direct transcriptional repression, in line with evidence that boosting DOXP/MEP supply enhances carotenoids, whereas *HMGR* mainly impacts sterols [36].

*Yellow flesh* (*r*)—*global output restriction with limited compensation.* In *r*, loss of *PSY1* resulted in a strong reduction of carotenoids at Br and FR, with lutein remaining relatively predominant. While transcriptional responses included transient up-regulation of *PSY2* at Br, metabolite levels were not restored, arguing against sustained transcriptional compensation [37]. This pattern indicates that pathway output is primarily constrained by precursor limitation, with only partial and stage-restricted regulatory responses. Consistent with this, downstream processes such as apocarotenoid formation remain substrate-limited [38].

*Tangerine* (*t*)—*stage-dependent uncoupling at the isomerization node*. In *t*, loss of *CrtISO1* caused stage-dependent alterations in carotenoid partitioning, with accumulation of upstream intermediates and reduced levels of downstream products at late ripening stages. While transcriptional changes were evident at MG, metabolite profiles at Br and FR were not matched by proportional gene expression, indicating increasing constraint downstream of the isomerization step. The persistence of low amounts of all-trans-lycopene likely reflects a combination of partial allele leakiness, non-enzymatic *cis*/*trans* isomerization, and residual or alternative isomerase activities [39], but overall supports a model in which pathway output is primarily constrained at the metabolic level rather than sustained by transcriptional regulation.

*Beta* (*B*)—*Enhanced β-cyclization and compensatory upstream activation*. In B, early induction of *CYC-b* redirected flux toward β–β– branch products, leading to depletion of linear intermediates and lycopene. This induction was already evident at MG, earlier than expected for a chromoplast-associated function [13]. Despite strong upregulation of early pathway genes at FR, upstream intermediates were not restored, indicating a compensatory transcriptional response that fails to recover metabolic output. This uncoupling between gene expression and metabolite levels suggests that pathway behavior is primarily constrained by precursor availability and flux redistribution rather than sustained transcriptional control. This interpretation is consistent with *LCY-b* overexpression studies [40], while multiplex *LCY* editing confirms branch sensitivity without resolving individual contributions [41].

*Delta* (*Del*)—*ε-branch reinforcement coupled to upstream repression and enhanced turnover.* In *Del*, increased ε-cyclization progressively redirected flux toward δ-carotene and lutein, with depletion of linear intermediates and lycopene at Br and FR. Unlike *B*, this repartitioning was accompanied by down-regulation of early-pathway genes and coordinated depletion of β–β–xanthophylls, although this comparison should be interpreted cautiously given the different genetic backgrounds. Late-stage induction of *CCD1b* and *NCED* indicates enhanced cleavage activity, consistent with increased α-ionone formation and reduced lycopene-derived apocarotenoids previously reported in *Del* [37].

### 3.2. Carotenoid Lesions Re-Balance the ABA Network

Because ABA derives from carotenoid cleavage, alterations in carotenoid metabolism are expected to impact ABA biosynthesis [42]. However, our integrated analysis at the FR stage indicates that carotenoid mutations do not translate into uniform changes in ABA levels, but rather into genotype-specific reconfiguration of the ABA network, affecting biosynthesis, catabolism and signaling components. Across mutants, variation in carotenoid pools was associated with changes in NCED-dependent cleavage and downstream ABA metabolism, but these did not follow a simple substrate-driven pattern. Instead, modulation of ABA catabolites and conjugated forms points to active homeostatic control, suggesting that ABA levels are buffered rather than passively reflecting precursor availability [42,43].

Consistently, the signaling module was also differentially affected. Changes in PYL receptors and PP2C phosphatases indicate adjustments in ABA responsiveness, with PP2C induction likely reflecting feedback attenuation of signaling, while modulation of perception components may help maintain signaling competence under fluctuating biosynthetic input [44].

These observations are in line with previous studies showing that manipulation of carotenoid flux affects ABA-related traits in tomato fruit [19,22]. However, our stage-resolved analysis indicates that this relationship is not linear: ABA behavior emerges from the integration of precursor supply, metabolic buffering, and signaling adjustments, particularly at late ripening stages [43].

### 3.3. Cross-Talk Among Plastidial Isoprenoid Branches

Although tocochromanol and quinone pathways have been extensively engineered, their integration within the fruit isoprenoid network remains not fully resolved. Our dataset shows that carotenoid perturbations only partially propagate to other isoprenoid branches, with limited transcriptional changes and modest, genotype-specific metabolic responses, indicating substantial pathway plasticity during ripening.

Within the established ripening framework [45], carotenoids act as a major sink for plastidial precursors such as GGPP. Reduced carotenoid flux is therefore associated with selective changes in other plastidial isoprenoids, as observed in *r*, where variation in tocochromanols and quinones at FR is accompanied by up-regulation of *HPPD* and *HPT*/*VTE2*, consistent with modulation of homogentisate-derived metabolism [46]. Despite this, total tocopherol levels were maintained or increased across genotypes, indicating that carotenoid perturbations do not reduce the output of competing branches and supporting the presence of metabolic buffering.

Additional buffering mechanisms likely involve chlorophyll turnover and recycling of phytyl chains into tocopherol biosynthesis [47], together with compensatory adjustments in *at*, potentially linked to residual IDI activity and/or MEP–MVA interactions. This is further supported by partially uncoupled behavior between plastidial (tocochromanols, plastoquinone, phylloquinone) and extra-plastidial (ubiquinones) isoprenoid pools, indicating that inter-compartmental coupling contributes to, but does not strictly constrain, isoprenoid homeostasis.

Overall, these results indicate that carotenoid metabolism acts as a buffering node within the fruit isoprenoid network, with perturbations accommodated through selective metabolic adjustments and flux redistribution [48].

### 3.4. Correlation Analysis Reveals Isoprenoid System Rewiring During Ripening

To obtain a system-level view of how carotenoid mutations reshape isoprenoid metabolism, we integrated HCL, correlation matrices and network analyses across ripening stages. Within this framework, the observed associations are best interpreted as coordinated trends, rather than direct regulatory relationships.

HCL provided a global readout of genotype relationships within the isoprenoid space. Despite possible background effects due to the distinct genetic background of *Del* (M82), genotype proximity at MG and Br suggests a shared metabolic framework buffering mutation-specific effects during early ripening, whereas at FR this structure is partially reorganized, with *t* diverging more clearly [49,50].

The correlation matrix further highlights a progressive restructuring of carotenoid coordination during ripening [51]. At MG, coordination is mainly observed at the transcriptional level, particularly among desaturation/isomerization genes, consistent with a system still resembling photosynthetic tissue. At Br, coordinated accumulation of carotenoids is accompanied by the emergence of opposing transcriptional modules (desaturation/isomerization versus cyclization), suggesting a balance between precursor throughput and branch allocation. At FR, correlations become more segregated, with distinct co-varying groups corresponding to early intermediates, terminal xanthophylls, and a partially independent lycopene pool.

Within this framework, lycopene and its isomers appear to act as a central buffering node, integrating upstream precursor supply with downstream branch utilization. The closer association between the lycopene pool and the ε–β– branch, compared to β–β– xanthophylls, is consistent with additional constraints imposed by ABA biosynthesis on the latter via NCED-mediated cleavage [43,52,53]. The progressive segregation of metabolic modules and the emergence of a central lycopene-associated pool at late ripening further support a model in which pathway coordination is maintained through dynamic, stage-dependent buffering mechanisms.

Correlation networks enabled visualization of coordinated gene–metabolite relationships beyond single-feature analyses. Hub-centered views around mutational targets further revealed how each lesion reshapes local interaction patterns across ripening stages [54].

At MG, isoprenoid-related hub formation becomes first detectable in our dataset, indicating that network assembly precedes the major metabolic transitions observed at Br and FR. Because MG can encompass distinct physiological sub-stages, finer staging resolution (e.g., early- vs. late-MG) [55] may further refine hub detection and improve the temporal and spatial interpretability of network-based analyses [56].

As ripening progresses, connectivity becomes increasingly structured. At FR, hub-centered networks highlight a highly coordinated module centered on *PSY1* (in *r*) and the fruit-specific lycopene β-cyclase *CYC-B* (in *Beta*), whose exclusively positive interactions suggest sustained transcriptional–metabolic coupling around both pathway entry and branch routing at late ripening stages. This pattern suggests that, even at full maturity, transcriptional–metabolic coupling remains tightly organized around both pathway entry (*PSY1*) and branch routing (β-cyclization via *CYC-b*), indicating that these steps continue to participate in shaping carotenoid flux at late ripening stages. In line with this interpretation, *CYC-b* displays a progressive increase in connectivity from MG to FR, consistent with an increasingly central role as ripening proceeds.

Lycopene β-cyclization has been linked to ripening and post-harvest traits. Fruit-specific *LCY-b* overexpression was reported to increase ABA levels and to delay softening/extend shelf life [19], whereas β-cyclase-dependent improvements in shelf life have also been discussed in frameworks that implicate additional (not strictly ABA-limited) metabolic layers [23]. One plausible—but still insufficiently tested—mechanistic layer is carotenoid-derived signaling beyond ABA, including apocarotenoids, which can act as bioactive regulators rather than mere cleavage by-products [57,58].

In parallel, *PSY1* is widely regarded as a major rate-controlling step in the tomato fruit carotenoid pathway, and its induction is a key driver of the ripening-associated carotenoid burst across species [59,60], influencing also the ABA levels in tomato fruit [61].

Consistent with this possibility, our hub-centered networks place *PSY1* and *CYC-b* in a tightly coordinated module (r = 0.92) and connect both nodes to ABA and multiple genes/metabolites of the ABA biosynthetic/signaling route. While these associations do not establish causality, they nominate a concrete hypothesis: coordinated “push–pull” modulation at *PSY1* (push) and β-cyclization via *CYC-b*/*LCYb* (pull) may be an effective entry point to dissect how carotenoid flux partitioning interfaces with ABA-linked (and potentially apocarotenoid-linked) control of late ripening and post-harvest behavior.

In contrast, *CrtISO1* (*t*) consistently exhibits a prevalence of negative interactions, reinforcing its association with a regulatory configuration linked to pathway restraint or re-balancing. In addition to negative correlations with carotenoid metabolites, *CrtISO1* shows inverse associations with genes involved in ABA, chlorophylls and MVA biosynthesis, pointing to a broader cross-talk between carotenoid desaturation, hormonal metabolism and upstream isoprenoid supply. This recurrent early negative connectivity suggests that *CrtISO1* may be embedded in a regulatory module coordinating multiple isoprenoid branches, and identifies *AAO*, *GSAAT*, *UROD* and *HMGS* as potential candidate nodes for future functional validation aimed at disentangling causal relationships within this network [62,63,64].

## 4. Materials and Methods

### 4.1. Plant Growth and Sampling

Wild-type (cv Ailsa Craig (AC) and M82) and tomato mutants (*Solanum lycopersicum* L.) were obtained from the Tomato Genetics Resource Center (TGRC, Davis, CA, USA), except for *Beta*, kindly provided by Prof. James Giovannoni (BTI, Ithaca, NY, USA). Genotype information and affected steps in carotenoid biosynthesis are reported in Figure 1.

Plants were grown under controlled conditions (16 h light/8 h dark; 25/20 °C day/night) in a randomized block design. Fruits were collected at mature green (MG), breaker (Br), and full ripe (FR) stages. For each genotype and stage, at least three biological replicates consisting of independent pools of fruits were collected, frozen in liquid nitrogen, and stored at −80 °C.

### 4.2. Genomic Analyses

Gene annotations and chromosomal positions were retrieved from the Sol Genomics Network. Genomic DNA was extracted from leaf tissue (DNeasy Plant Mini Kit, Qiagen, Hilden, Germany). Candidate genes were amplified by PCR and sequenced (ABI 3730 DNA Analyzer, Applied Biosystems, Foster City, CA, USA). Sequence assembly and alignment were performed using CodonCode Aligner (v.10.0.2; CodonCode Corporation, Centerville, MA, USA) and Clustal Omega (EMBL-EBI, https://www.ebi.ac.uk/Tools/msa/clustalo/, accessed on 11 May 2026). Promoter regions (2 kb upstream of ATG) were analyzed for *cis*-elements as previously described [65]. Functional effects of sequence variants were predicted using SIFT (https://sift.bii.a-star.edu.sg/; accessed on 11 May 2026) and PROVEAN (https://provean.jcvi.org/; accessed on 11 May 2026).

### 4.3. Gene Expression Analyses

RNA extraction and QRT-PCR analyses were performed as previously described [19,66]. Gene-specific primers are listed in Table S1. Expression data were normalized to actin and expressed relative to the corresponding wild-type. Statistical analysis (one-way ANOVA followed by Tukey’s test) was performed using PAST software (https://past.en.lo4d.com/windows, accessed on 11 May 2026).

### 4.4. Metabolomic Analyses

Extraction and LC-based analyses of carotenoids and related isoprenoids were performed as previously described [67]. Briefly, metabolites were extracted from lyophilized pericarp tissue and analyzed using an LTQ-Orbitrap Discovery mass spectrometer (Thermo Fisher Scientific Waltham, MA, USA) coupled to an Accela UHPLC system, operating in negative APCI mode. Carotenoids were detected using a photodiode array detector (220–700 nm) and quantified using external standards or published extinction coefficients, normalized to an internal standard. Compound identification was based on comparison with authentic standards and accurate mass information, as previously reported [10]. ABA and related metabolites were analyzed as described in [19]. For each genotype, at least three biological replicates and two technical replicates were analyzed.

### 4.5. Microarray Analyses

Microarray experiments were carried out using EU-TOM3 platform (Affymetrix, Santa Clara, CA, USA) and an external service provided by IFOM (Fondazione Istituto FIRC di Oncologia Molecolare—COGENTECH, Milan, Italy) as previously described [19]. Microarray data have been deposited in the GEO public repository (https://www.ncbi.nlm.nih.gov/geo, accessed on 13 April 2026) under the accession number GSE327902.

### 4.6. Bioinformatics

Custom carotenoid and ABA metabolic map were built as previously described [19,68]. Hierarchical clustering (HCL), correlation matrices and correlation networks were generated as previously described [69].

### 4.7. Statistical Analysis

Statistical significance between each mutant genotype and the corresponding wild-type at the same developmental stage was assessed using two-tailed Student’s *t*-tests. For each metabolite or transcript, comparisons were performed independently at each ripening stage. Data are presented as mean ± SD of biological replicates. Differences were considered statistically significant at *p* < 0.05.

## 5. Conclusions

By characterizing five tomato mutants affecting distinct steps of the carotenoid pathway, we show that carotenoid metabolism remains active throughout fruit development, even when major enzymatic nodes such as *PSY1* or *CrtISO1* are genetically perturbed. Rather than producing strictly local effects, these lesions are associated with coordinated changes across distant pathway segments and across plastidial isoprenoid groups, highlighting a high degree of metabolic buffering.

A first key outcome is the frequent uncoupling between transcriptional variation and metabolite output, indicating that pathway behavior cannot be inferred from gene expression alone but reflects the combined effects of precursor availability, flux redistribution and downstream constraints (post-transcriptional and post-translational regulation, feedback effects).

A second key feature is the central role of flux distribution within the pathway. The two cyclization branches behave in a coordinated manner, with changes in one mirrored by changes in the other. Network analysis highlights pathway entry and branch points as the main control nodes, while *CrtISO1* appears to contribute to pathway balancing rather than driving flux.

A third key aspect is the strong stage dependency of the system. Genotype-specific differences are already evident at early ripening stages, but become more pronounced and structured at full ripening, indicating a progressive shift in regulatory control during chromoplast development.

Overall, our integrated metabolite–transcript profiling and correlation-based network analyses define carotenoid metabolism as a central buffering node within the fruit isoprenoid network, where genetic perturbations are accommodated through metabolic plasticity and cross-pathway coordination rather than extensive transcriptional reprogramming.

## Data Availability

Affymetrix array data have been deposited in the NCBI Gene Expression Omnibus (GEO) under accession number GSE327902.

## Supplementary Materials

The following supporting information can be downloaded at: https://www.mdpi.com/article/10.3390/ijms27104412/s1.

## Abbreviations

The following abbreviations are used in this manuscript:

**Acronym**	**Meaning**
MVA	mevalonate pathway
MEP	methylerythritol 4-phosphate pathway
IPP	isopentenyl diphosphate
DMAPP	dimethylallyl diphosphate
GPP	geranyl diphosphate
FPP	farnesyl diphosphate
GGPP	geranylgeranyl diphosphate
CAR	carotenoids
PSY1	phytoene synthase 1
PDS	phytoene desaturase
ZISO	ζ-carotene isomerase
ZDS	ζ-carotene desaturase
CrtISO	carotenoid isomerase
LCY-b	lycopene β-cyclase
LCY-e	lycopene ε-cyclase
CYC-b	chromoplast-specific lycopene β-cyclase
CHY	β-carotene hydroxylase
LUT1	carotenoid ε-ring hydroxylase (CYP97C)
LUT5	β-carotene hydroxylase (CYP97A)
CCD	carotenoid cleavage dioxygenase
NCED	9-cis-epoxycarotenoid dioxygenase
CHL	chlorophylls
CHLI	magnesium chelatase subunit I
CHLD	magnesium chelatase subunit D
CHLH	magnesium chelatase subunit H
CHLM	Mg-protoporphyrin IX methyltransferase
CRD1	Mg-protoporphyrin IX monomethyl ester cyclase
POR	protochlorophyllide oxidoreductase
CAO	chlorophyllide a oxygenase
CHLG	chlorophyll synthase
CHLP	geranylgeranyl reductase
TOC	tocochromanols
VTE1	tocopherol cyclase
VTE2	homogentisate phytyltransferase
VTE3	2-methyl-6-phytyl-1,4-benzoquinol methyltransferase
VTE4	γ-tocopherol methyltransferase
QUI	quinones
MEN	menaquinone biosynthesis enzymes
UbiE	methyltransferase involved in quinone biosynthesis
DHNA	1,4-dihydroxy-2-naphthoate
MG	mature green stage
Br	breaker stage
FR	red ripe stage
PCA	principal component analysis
OPLS-DA	orthogonal partial least squares discriminant analysis
VIP	variable importance in projection
r	Pearson correlation coefficient
HCL	hierarchical clustering
ns	node strength
