# Supplementary material for: Decoding Isoprenoid Transcript–Metabolite Interactions in Carotenoid Tomato Fruit Mutants Uncovers Novel Metabolic Cross-Links
Source: Int J Mol Sci. 2026 May 15;27(10):4412. doi: 10.3390/ijms27104412 (PMC13207119; doi:10.3390/ijms27104412)
Supplement: Supplementary file 1 [file ijms-27-04412-s001.zip › Supplemental materials_Frusciante et al., 2026/Supp_Figures_Frusciante et al., 2026 copia.pptx]

## Slide 1
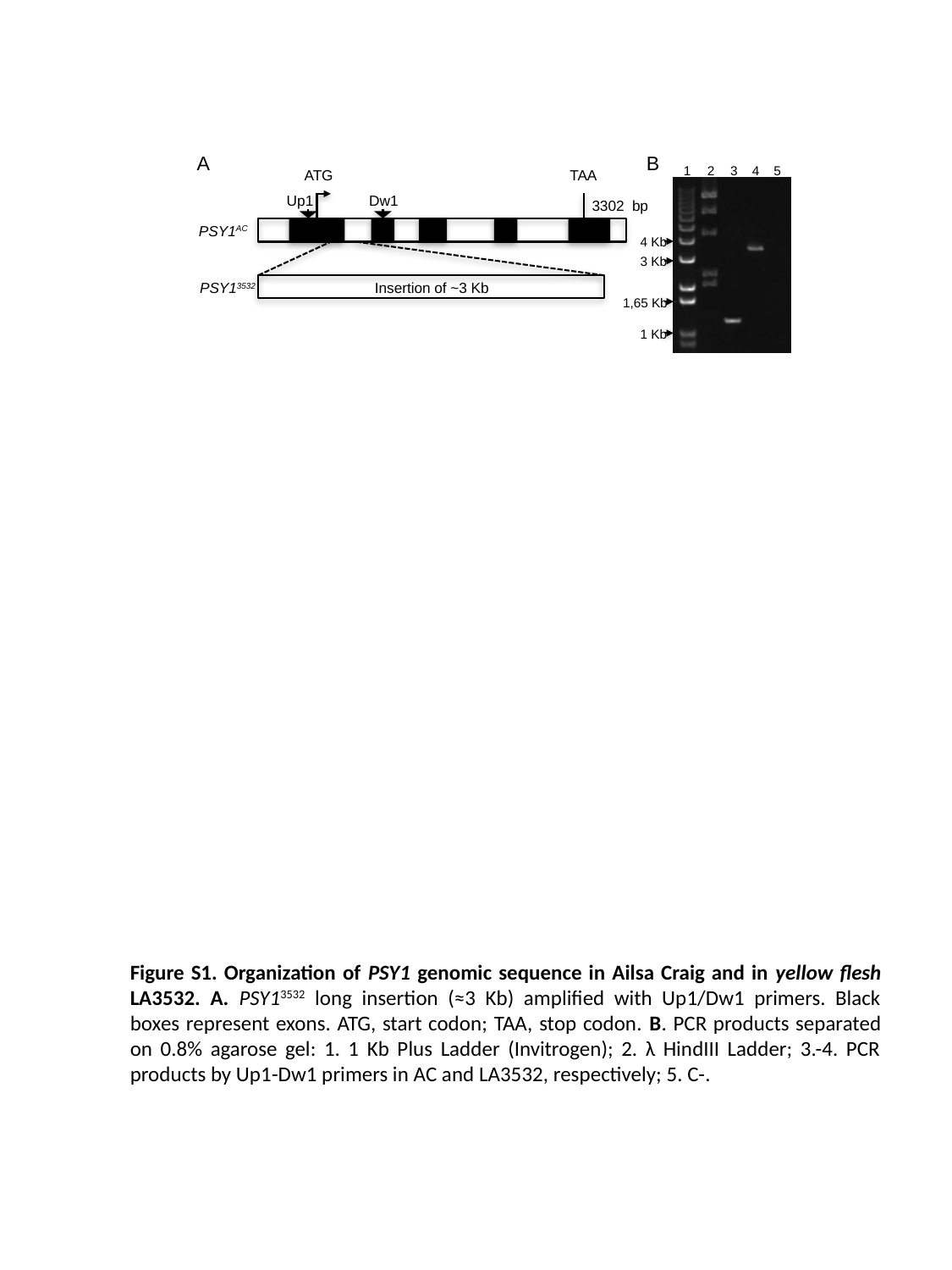

A
B
1
2
3
4
5
4 Kb
3 Kb
1,65 Kb
1 Kb
ATG
TAA
Up1
Dw1
3302 bp
PSY1AC
PSY13532
Insertion of ~3 Kb
Figure S1. Organization of PSY1 genomic sequence in Ailsa Craig and in yellow flesh LA3532. A. PSY13532 long insertion (≈3 Kb) amplified with Up1/Dw1 primers. Black boxes represent exons. ATG, start codon; TAA, stop codon. B. PCR products separated on 0.8% agarose gel: 1. 1 Kb Plus Ladder (Invitrogen); 2. λ HindIII Ladder; 3.-4. PCR products by Up1-Dw1 primers in AC and LA3532, respectively; 5. C-.

## Slide 2
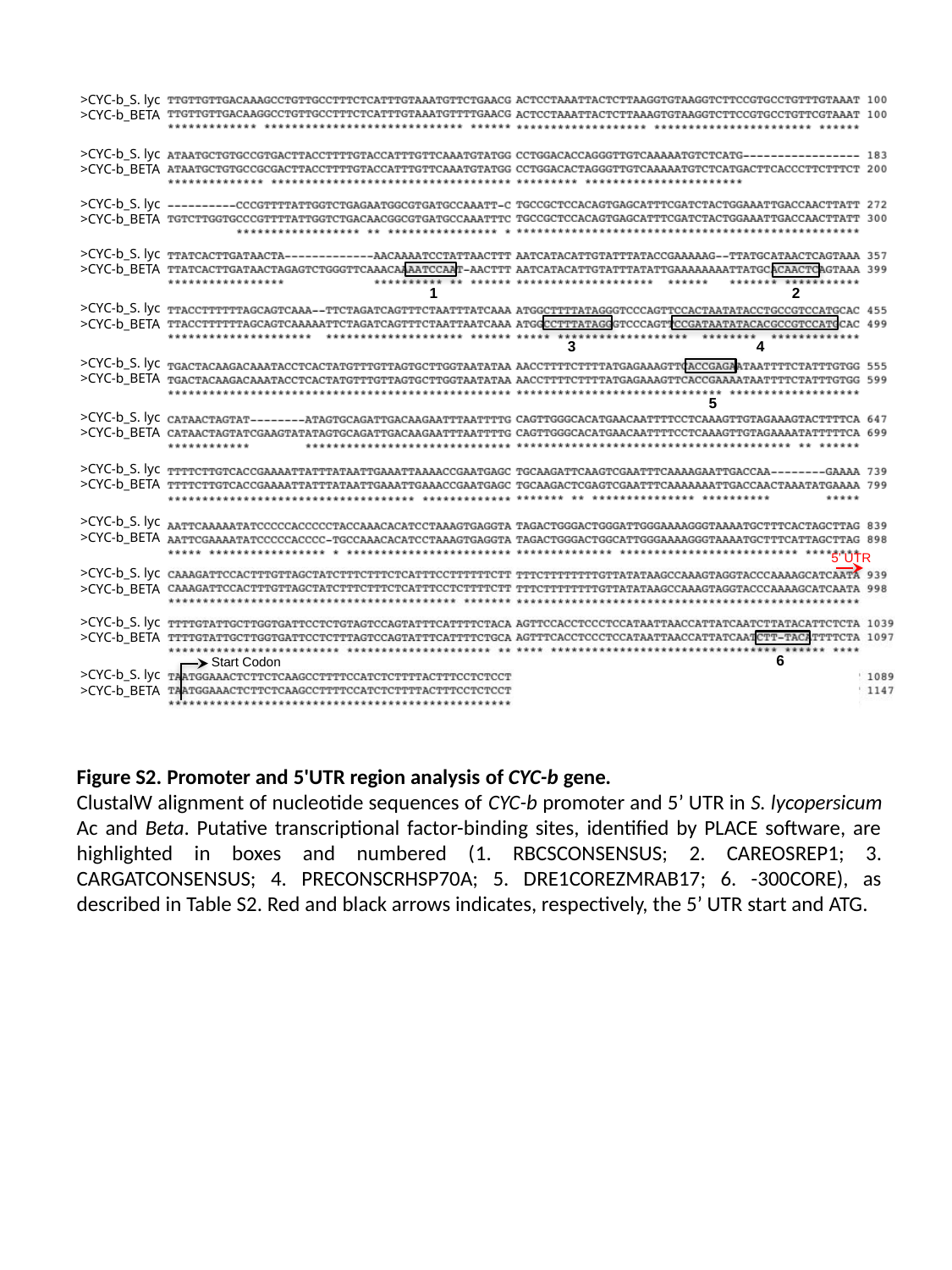

>CYC-b_S. lyc
>CYC-b_BETA
>CYC-b_S. lyc
>CYC-b_BETA
>CYC-b_S. lyc
>CYC-b_BETA
>CYC-b_S. lyc
>CYC-b_BETA
>CYC-b_S. lyc
>CYC-b_BETA
>CYC-b_S. lyc
>CYC-b_BETA
>CYC-b_S. lyc
>CYC-b_BETA
>CYC-b_S. lyc
>CYC-b_BETA
>CYC-b_S. lyc
>CYC-b_BETA
>CYC-b_S. lyc
>CYC-b_BETA
>CYC-b_S. lyc
>CYC-b_BETA
>CYC-b_S. lyc
>CYC-b_BETA
1
2
3
4
5
6
5’ UTR
Start Codon
Figure S2. Promoter and 5'UTR region analysis of CYC-b gene.
ClustalW alignment of nucleotide sequences of CYC-b promoter and 5’ UTR in S. lycopersicum Ac and Beta. Putative transcriptional factor-binding sites, identified by PLACE software, are highlighted in boxes and numbered (1. RBCSCONSENSUS; 2. CAREOSREP1; 3. CARGATCONSENSUS; 4. PRECONSCRHSP70A; 5. DRE1COREZMRAB17; 6. -300CORE), as described in Table S2. Red and black arrows indicates, respectively, the 5’ UTR start and ATG.

## Slide 3
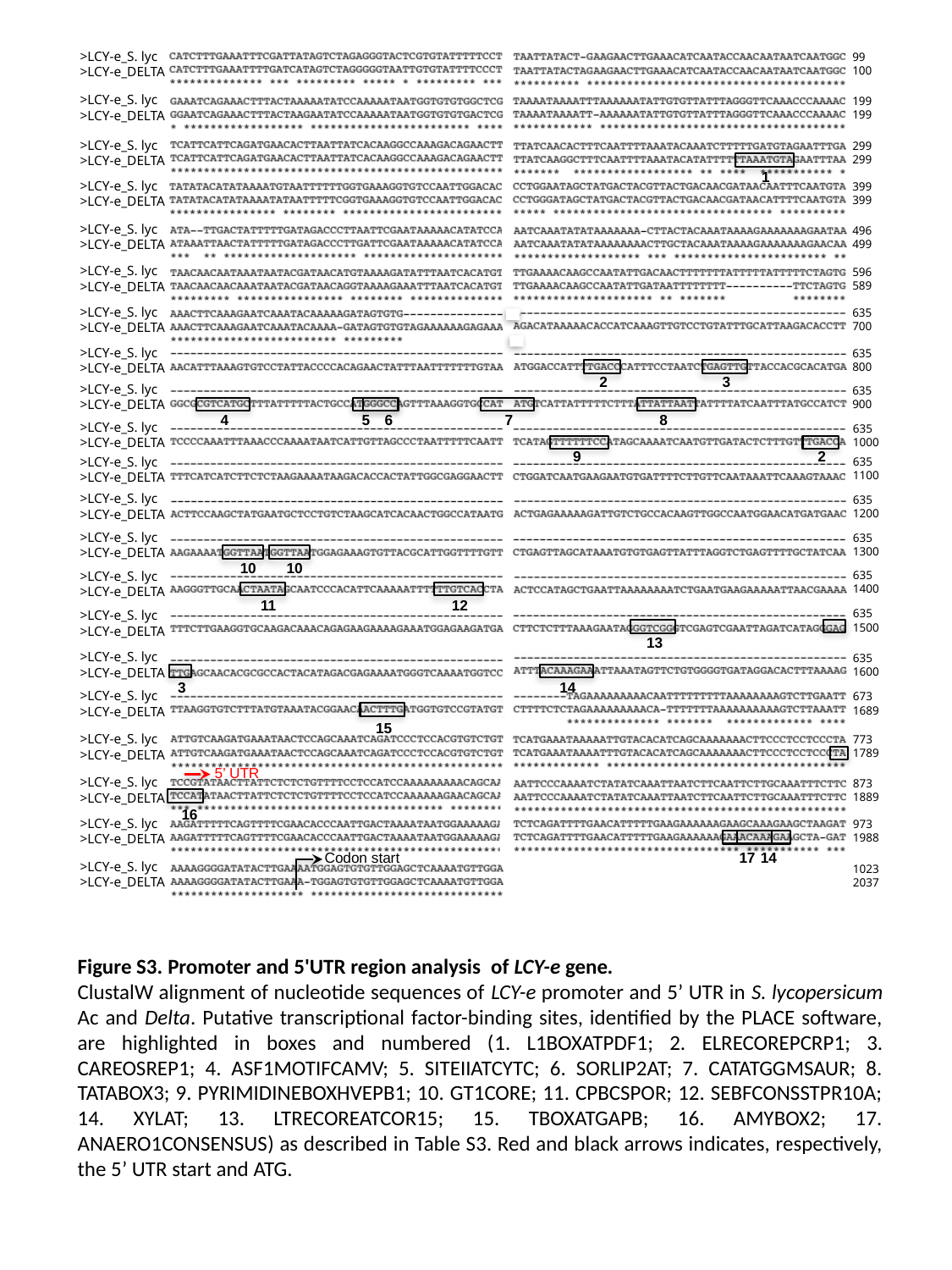

>LCY-e_S. lyc
>LCY-e_DELTA
>LCY-e_S. lyc
>LCY-e_DELTA
>LCY-e_S. lyc
>LCY-e_DELTA
>LCY-e_S. lyc
>LCY-e_DELTA
>LCY-e_S. lyc
>LCY-e_DELTA
>LCY-e_S. lyc
>LCY-e_DELTA
>LCY-e_S. lyc
>LCY-e_DELTA
>LCY-e_S. lyc
>LCY-e_DELTA
>LCY-e_S. lyc
>LCY-e_DELTA
>LCY-e_S. lyc
>LCY-e_DELTA
>LCY-e_S. lyc
>LCY-e_DELTA
>LCY-e_S. lyc
>LCY-e_DELTA
>LCY-e_S. lyc
>LCY-e_DELTA
>LCY-e_S. lyc
>LCY-e_DELTA
>LCY-e_S. lyc
>LCY-e_DELTA
>LCY-e_S. lyc
>LCY-e_DELTA
>LCY-e_S. lyc
>LCY-e_DELTA
>LCY-e_S. lyc
>LCY-e_DELTA
>LCY-e_S. lyc
>LCY-e_DELTA
>LCY-e_S. lyc
>LCY-e_DELTA
>LCY-e_S. lyc
>LCY-e_DELTA
99
100
199
199
299
299
1
399
399
496
499
596
589
635
700
635
800
2
3
635
900
5
6
7
8
635
1000
9
2
635
1100
635
1200
635
1300
10
10
635
1400
11
12
635
1500
13
635
1600
3
14
673
1689
15
773
1789
873
1889
16
973
1988
17
14
1023
2037
4
5’ UTR
Codon start
Figure S3. Promoter and 5'UTR region analysis of LCY-e gene.
ClustalW alignment of nucleotide sequences of LCY-e promoter and 5’ UTR in S. lycopersicum Ac and Delta. Putative transcriptional factor-binding sites, identified by the PLACE software, are highlighted in boxes and numbered (1. L1BOXATPDF1; 2. ELRECOREPCRP1; 3. CAREOSREP1; 4. ASF1MOTIFCAMV; 5. SITEIIATCYTC; 6. SORLIP2AT; 7. CATATGGMSAUR; 8. TATABOX3; 9. PYRIMIDINEBOXHVEPB1; 10. GT1CORE; 11. CPBCSPOR; 12. SEBFCONSSTPR10A; 14. XYLAT; 13. LTRECOREATCOR15; 15. TBOXATGAPB; 16. AMYBOX2; 17. ANAERO1CONSENSUS) as described in Table S3. Red and black arrows indicates, respectively, the 5’ UTR start and ATG.

## Slide 4
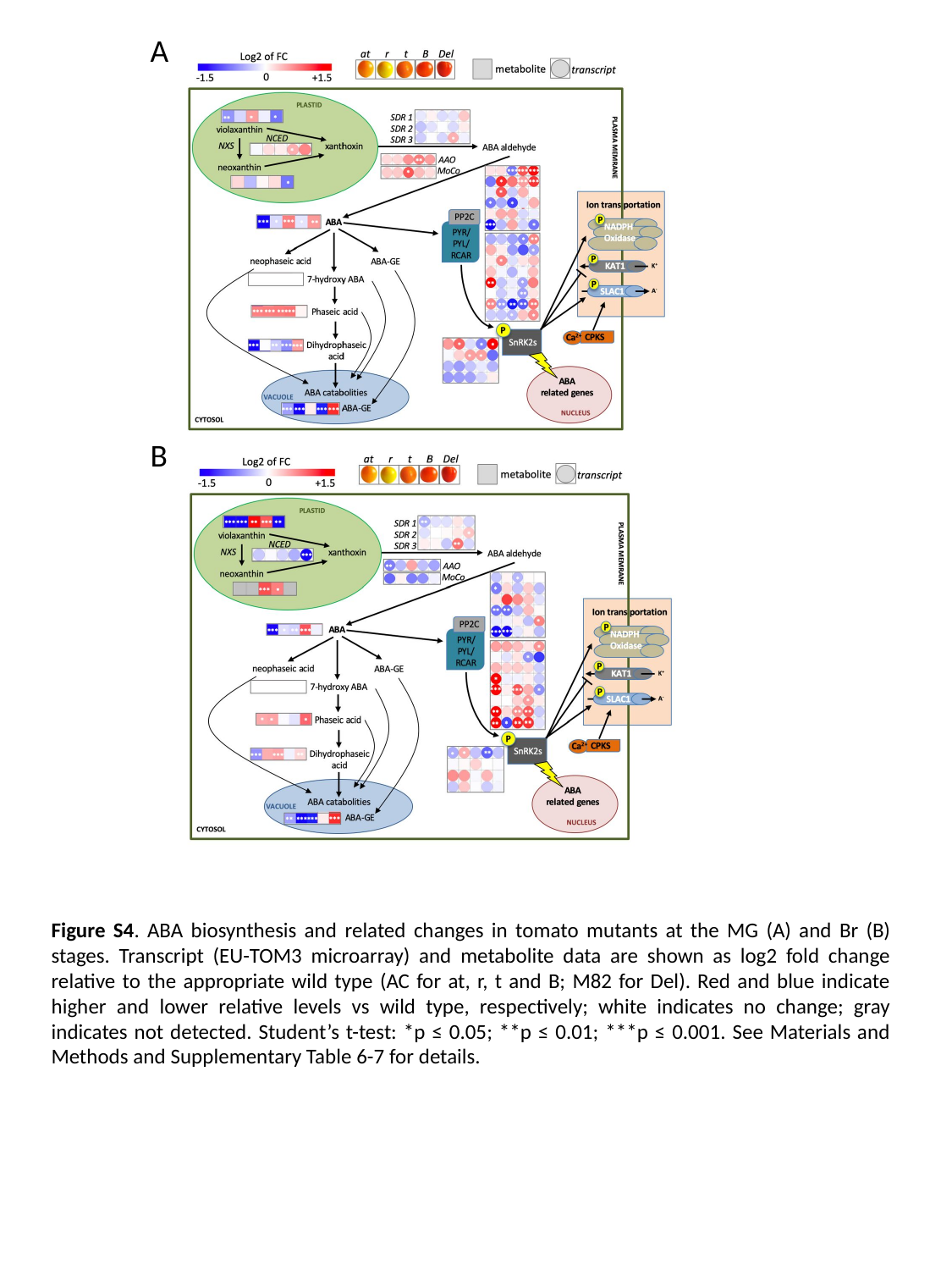

A
B
Figure S4. ABA biosynthesis and related changes in tomato mutants at the MG (A) and Br (B) stages. Transcript (EU-TOM3 microarray) and metabolite data are shown as log2 fold change relative to the appropriate wild type (AC for at, r, t and B; M82 for Del). Red and blue indicate higher and lower relative levels vs wild type, respectively; white indicates no change; gray indicates not detected. Student’s t-test: *p ≤ 0.05; **p ≤ 0.01; ***p ≤ 0.001. See Materials and Methods and Supplementary Table 6-7 for details.

## Slide 5
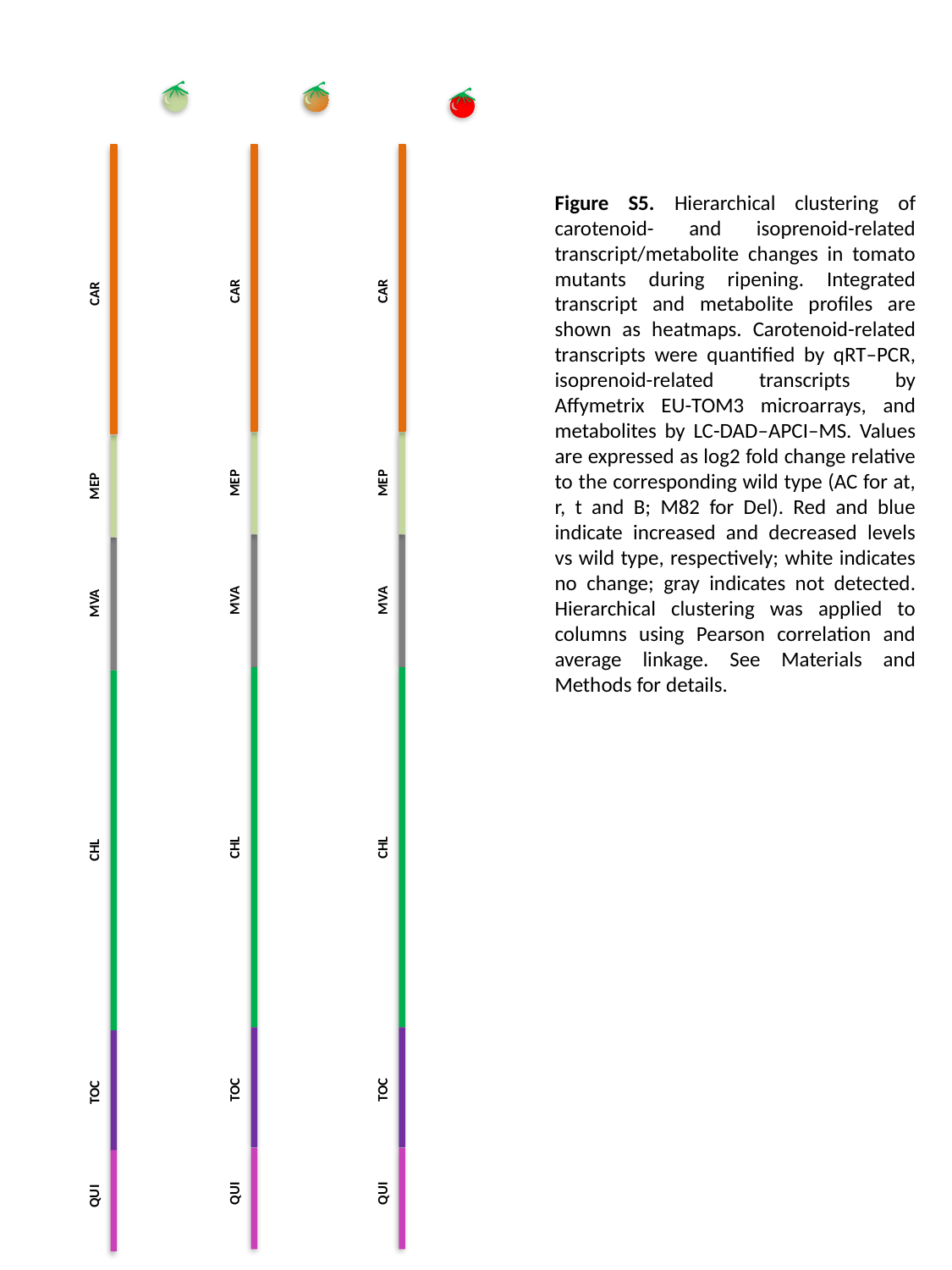

Figure S5. Hierarchical clustering of carotenoid- and isoprenoid-related transcript/metabolite changes in tomato mutants during ripening. Integrated transcript and metabolite profiles are shown as heatmaps. Carotenoid-related transcripts were quantified by qRT–PCR, isoprenoid-related transcripts by Affymetrix EU-TOM3 microarrays, and metabolites by LC-DAD–APCI–MS. Values are expressed as log2 fold change relative to the corresponding wild type (AC for at, r, t and B; M82 for Del). Red and blue indicate increased and decreased levels vs wild type, respectively; white indicates no change; gray indicates not detected. Hierarchical clustering was applied to columns using Pearson correlation and average linkage. See Materials and Methods for details.
CAR
CAR
CAR
MEP
MEP
MEP
MVA
MVA
MVA
CHL
CHL
CHL
TOC
TOC
TOC
QUI
QUI
QUI
